# To the Editors of the Medical and Physical Journal

**Published:** 1800-04

**Authors:** H. Davies

**Affiliations:** Piccadilly


### *To the Editors of the Medical and Phyfical Journal.*

GENTLEMEN,

CONCEIVING your correfpondent, Mr. PECK, to have mifapprehended the nature of my cafe, on "the adhefion of the placenta," noticed by him in the laft Journal; by inferting the annexed elucidation of it, you will much oblige,

GENTLEMEN,

Your's, refpectfully,

H. DAVIES.

*Piccadilly,*
*March* 7, 1800.

---

THAT the placenta, in all cafes where no difficulty occurs, fhould be *early* delivered, is a fentiment perfectly confonant with found practice. Nature alone, when unaffifted by Art, points out the propriety of it, by its fpeedy expulfion after the birth of the fœtus. But as it is retained in utero, now and then, by various caufes, it behoves the practitioner to adapt his mode of treatment, as nearly as he can, to the nature of the circumftance occafioning the retention.

Mr. Peck has only mentioned two caufes of its retention, viz. "the rupture of the funis, and the irregular contraction of the uterus." I think a *third* fhould have been added, which, in my opinion, is far more formidable than either of the former, namely, *a firm adhefion of a confiderable part of the placenta to the internal furface of the uterus*; which is not unfrequently the cafe, and not eafily detached without either endangering an inverfion, if forcibly attempted, or producing fymptoms of irritation, if long continued; and thofe of fo alarming a nature, that the patient has been known to *fink* under the operation; of which melancholy circumftance, I was once a painful fpectator.

This was, in a great meafure, the ftate of my patient's cafe, which Mr. P. condefcended to comment upon; with whom I am by no means difpleafed, but, on the contrary, obliged for what he conceived to be ufeful hints. Yet, as fome theories, rigidly perfifted in, will not apply in all cafes, a deviation, therefore, from eftablifhed rules, under certain circumftances, will not only be juftifiable, but indifpenfable.

I mentioned that the *exhauftion* of my patient was the caufe of defifting from my attempt to feparate the placenta then. Mr. P. inquires into the *caufe* of the exhauftion; and replies, that it was the hæmorrhage; from whom I beg leave to differ, and do not hefitate to affirm, it was owing to the *fatigue* occafioned

ſioned by the previous labour, together with the irritation produced in endeavouring to detach the placenta from its adheſion to the uterus; for no material hæmorrhage had taken place *at that time*, which I thought fully juſtified me in giving the patient a longer reſpite; and when I found, after waiting a while, its tendency to *increaſe*, I proceeded to remove the cauſe, by extracting as much as I could of it with ſafety, leaving the remainder to the powers of the ſyſtem, invigorated by the plan I hinted at in the relation of the caſe, which fully anſwered my expectations; and, if a ſimilar caſe occurs in the courſe of my practice, I ſhould not think myſelf warranted to proceed upon any different plan.

Having ſaid thus much in vindication of the practice, I now diſmiſs the ſubject, taking it for granted, that every practitioner will be ſtudious to adopt that mode of treatment, which will appear to be moſt conducive to the welfare of his patient, as well as his own reputation. And, as a difference of opinion will more or leſs obtain upon moſt ſubjects, it is but candid to hope that this very difference may ultimately tend to general utility.

---

